# Iodine for the Outpatient Management of Graves’ Disease: A Case Series of 7 Patients

**DOI:** 10.1016/j.aed.2025.10.015

**Published:** 2025-10-27

**Authors:** Oyunbileg Magvanjav, Ryan S. MacLeod, Priyadarshini Balasubramanian, Kavya Mekala, Patricia R. Peter, Silvio E. Inzucchi, Sachin K. Majumdar

**Affiliations:** Section of Endocrinology and Metabolism, Yale University School of Medicine, New Haven, Connecticut

**Keywords:** Graves’ disease, hyperthyroidism, iodine, methimazole, potassium iodide, thionamide

## Abstract

**Background/Objective:**

Methimazole (MMI) and propylthiouracil are common treatments for Graves’ disease (GD), but few options exist for patients who cannot tolerate these drugs or prefer to avoid radioactive iodine or surgery. Iodine is an alternative treatment but is infrequently used due to concerns of transient effectiveness.

**Case Description:**

Seven patients (ages 24-77, mean 53.7 years) with GD previously on thionamides received iodine (saturated solution of potassium iodide or Lugol’s 5% solution) for periods ranging from 3 weeks to over a year. Four patients started iodine for MMI-associated transaminitis, one for neutropenia on MMI and methotrexate, one for chemotherapy-induced pancytopenia, and one for uncontrolled hyperthyroidism on high-dose propylthiouracil. Doses of iodine ranged 60-750 mg/d; mean treatment duration: 134 ± 135 days. Five patients eventually underwent thyroidectomy, one patient with leukemia entered hospice, and one remains controlled on iodine monotherapy. Of the 7 patients, 71% maintained normal thyroid hormone levels and 29% had recurrence of hormone elevations.

**Discussion:**

Iodine monotherapy can effectively control GD for as long as 1 year. Compared to thionamides, iodine has a more favorable safety profile and may be a suitable option for patients who are unable or unwilling to undergo radioactive iodine or surgery, or for those who cannot tolerate thionamides. In select patients, iodine may serve as a bridging therapy to definitive treatments such as surgery.

**Conclusion:**

Iodine may offer a viable alternative for treating GD, particularly for situations where patients cannot tolerate thionamides or have a contraindication, prefer alternative nonthionamide therapy, or as a bridge to surgery.

**Highlights**

• We describe our experience with iodide monotherapy for the outpatient management of Graves’ disease in 7 patients with treatment durations ranging from 1 month to over a year
• Disease control with iodine monotherapy can last over a year. Compared with thioamides, iodine may have a more favorable safety profile and may be appropriate for patients who are unable or unwilling to undergo radioactive iodine, surgery, or who cannot take thioamides. In these select patients, iodine could serve as a longer-term bridge to definitive treatments like surgery
• Our findings contribute to the broader clinical application of iodine for the treatment of Graves’ disease and can inform clinical practice and evidence-based guidelines

**Clinical Relevance**

Graves’ disease is routinely managed with thioamides; however, many patients experience side effects on these medicines and have to resort to thyroidectomy or radioactive iodine as last resort options. Iodine is an alternative with a safer side effect profile. We report on the feasibility of iodine monotherapy for the long-term management of Graves’ disease in select situations where thioamides, radioactive iodine, or surgery are not an option or not preferred.

## Introduction

In 1863, Armand Trousseau mistakenly prescribed tincture of iodine to a young woman with tachycardia and exophthalmic goiter, resulting in her heart rate lowering from 140-150 to 90 beats per minute in about 15 days.^1^ When he switched her to the intended treatment, tincture of digitalis, tachycardia recurred, prompting him to reintroduce iodine therapy.^1^ He cautioned that iodine is usually harmful in Graves’ disease (GD) and early reports note that improvement can be followed by a worsening of symptoms.^2^^,^^3^ Iodine has been used to prepare patients with GD for thyroid surgery ever since Henry Plummer’s original publication in 1923, but its use for the non-surgical treatment of GD was supplanted by thionamide drugs and radioactive iodine (RAI).^4^ Presently, the mainstays of pharmacotherapy for GD are methimazole (MMI) and propylthiouracil (PTU).^5^ Thionamides carry potential adverse effects, including rash, agranulocytosis, elevated liver enzymes, teratogenicity, and, rarely, fulminant liver failure (with PTU presenting a higher risk than MMI).^5^ For patients intolerant to thionamides, preferring to avoid surgery or RAI, choices are limited. Iodine may be an appropriate option for GD management in such circumstances.

Iodine can rapidly inhibit thyroid hormone production in the thyroid gland, both transiently, Wolff-Chaikoff effect, and chronically.^6^, ^7^, ^8^ Beginning with Plummer and others in the 1920-1930s, iodine has been recognized for its ability to control thyrotoxicosis. More recent studies from Japan show its safety and effectiveness for the longer-term management of GD.^9^ While preoperative iodine is still used for GD, its use as a primary therapy in the USA has been avoided due to limited experience and concerns for thyrotoxicosis.^5^ However, in some circumstances it appears useful. Herein, we describe 7 patients who were managed with iodine therapy for durations of 3 weeks to over 1 year.

## Case Presentation

We included 7 patients with GD, all previously treated with a thionamide, that were subsequently treated with oral iodine solutions. Four patients started iodine for MMI-associated transaminitis (patients #1, #2, #4, #5); one patient (patient #3) for pancytopenia during chemotherapy for acute leukemia—he had already been taking MMI, but discontinued it to avoid worsening of the pancytopenia; another patient for severe neutropenia on MMI + methotrexate (patient #6); and one patient (patient #7) already taking PTU 600 mg/d added iodine for uncontrolled hyperthyroidism despite the high-dose PTU. Iodine treatment refers to either saturated solution of potassium iodide (SSKI) 1 g/mL (1 drop = 50 mg of iodide) or Lugol’s 5% solution (combination of 5% elemental iodine and 10% potassium iodide, 1 drop = ∼5 mg of iodine). Table describes each patient. Ages ranged 24-77 years (mean 53.7 ± 22.9); 71% were female. On exam, 4 patients had moderate, 2 mild, and one no thyroid enlargement.

**Table:** Thyroid Function Tests on Iodine Therapy^a^: Treatment Regimen and Outcomes

Patient #	Age	Sex	Preiodine TSH	Preiodine FT4	Initial iodine dose	Days to normal TFT	Duration of iodine therapy	Final iodine dose	Final FT4	Final therapy/Outcome
	Years		mIU/mL	ng/dL	mg/d	Days	Days	mg/d	ng/dL	
			RR: 0.27-4.20	RR: 0.80-1.70					RR: 0.80-1.70	
1	28	F	<0.01	**2.90**	100 mg	14	398	100 mg	1.01	Surgery
2^b^	77	F	0.152	1.44	450 mg	4	74	150 mg	**3.73**	Surgery
3	75	M	<0.005	**4.68**	450 mg	8	38	60 mg	**1.96**	Hospice
4	65	F	<0.005	**1.83**	150 mg	27	41	150 mg	0.84	Surgery
5^c^	38	M	0.011	1.09	750 mg	7	20	750 mg	0.90	Surgery
6^d^	69	F	0.008	1.39	100 mg	35	191	100 mg	0.57	Remains on iodine
7^e^	24	F	<0.01	n.a.	300 mg	28	179	300 mg	1.00	Surgery

Abbreviations: F = female; FT4 = free T4; M = male; MMI = methimazole; n.a. = not applicable; PTU = propylthiouracil; RR = reference range; SSKI = saturated solution of potassium iodide; T3 = total T3; TFT = thyroid function test; TSH = thyroid stimulating hormone.

Note: The elevated FT4 levels are highlighted in bold. All patients were on MMI or PTU prior to switching to iodine, with the exception of patient #7, who continued PTU as an add-on to iodine.

a Iodine therapy refers to either SSKI or Lugol’s 5% solution. All patients were on SSKI throughout their iodine therapy, except for patients #3 and #4: patient #3 was started on SSKI 450 mg/d then switched to Lugol's 5% solution 0.2 mL three times daily (equivalent to 60 mg/d of iodine) at hospital discharge, and patient #4 was on Lugol's 5% solution 10 drops three times daily (equivalent to 150 mg/d of iodine).

b Preiodine, the FT4 was normal, but TSH was suppressed at 0.152 mIU/mL. Days to normal TFT represent days to first normal TSH.

c Preiodine, the FT4 was normal, and total T3 was elevated at 219 ng/dL (normalized to 115 ng/dL at the end of iodine therapy) (Reference range: 72-153 ng/dL). Here, days to normal TFT represent days to first normal or non-elevated total T3.

d Preiodine FT4 was normal, but TSH was suppressed at 0.008 mIU/mL. Days to normal TFT represent days to first normal TSH.

e Patient #7 required up to 600 mg daily of PTU before iodine was added on. The addition of iodine allowed for the lowering of the PTU dose to 250 mg daily. Pre-iodine, the FT4 for this patient was not tested. Instead, the total T3 was being followed. The preiodine T3 level was normal at 147 ng/dL and remained normal. Here, days to normal TFT represent days to first normal TSH.

All patients were on a thionamide prior to treatment with iodine. Regarding prior MMI duration, patient #1 was on MMI for 2 weeks (last dose: 20 mg/d); patient #2 for 1.5 months (5 mg/d); patient #3 for 6.5 years (5 mg/d); patient #4 for 2 months (15 mg/d); patient #5 for 3 months (10 mg/d); and patient #6 for 3 months (5 mg/d). Patient #7 was on MMI for 2 months before switching to PTU due to myalgia and remained on PTU for 2.5 years (last dose: 250 mg/d). Initially, she was on PTU 600 mg/d, but this was reduced to 250 mg/d after adding iodine.

Iodine treatment duration ranged 20-398 days (mean ± SD: 134.4 ± 135.0, median: 74, interquartile range (IQR): 38-191). Hormone levels were monitored on average every 11 days during the first 2 months, then approximately every 2 months thereafter. Five patients received SSKI, and 2 received Lugol’s 5% solution. Initial SSKI doses ranged from 100-750 mg/d; Lugol’s 5% solution doses ranged from 60 to 150 mg/d. The overall mean starting iodine dose was 328.6 ± 239.5 mg/d (median: 300; IQR: 125-450).

Individual FT4 responses over time on iodine therapy are shown in Fig. Pre-iodine, the mean initial free T4 (FT4) level was 2.2 ± 1.4 ng/dL (reference: 0.8-1.7 ng/dL). Of the 7 patients, 3 had elevated FT4 (patients #1, #3, #4); 3 had normal FT4 (patients #2, #5, #6), of which one had elevated T3 (patient #5); and one had suppressed thyroid stimulating hormone (TSH) only (patient #7).

**Fig:**
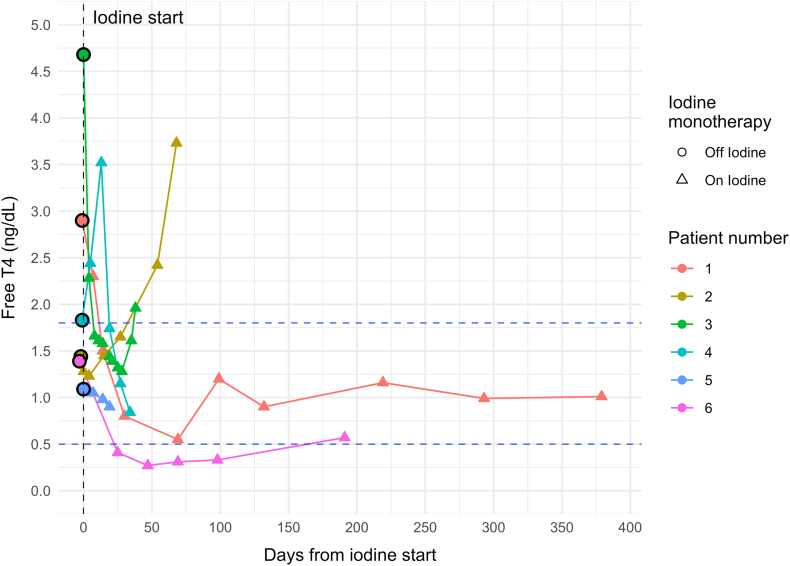
Free T4 levels preiodine and over time on iodine. Note: Patient #1-6 are shown; patient #7 was not included as only T3 levels were being followed. All 6 patients were on methimazole prior to switching to iodine monotherapy due to an adverse side effect or contraindication to methimazole. The plot shows free T4 levels from before the start of iodine treatment (SSKI or Lugol’s 5% solution) until the last laboratory free T4 test before thyroidectomy, or until follow-up in patients who did not undergo thyroidectomy (patients #2 and #6). Dashed horizontal lines indicate *upper* and *lower* limits of reference free T4 range (0.80 - 1.70 ng/dL). SSKI = saturated solution of potassium iodide.

After iodine initiation, the 3 patients with previously elevated FT4 (patients #1, #3, #4) responded well: their mean time to FT4 normalization was 16.3 ± 9.7 days (median: 14; IQR: 8-27). Patient #5 eventually normalized T3 (from 219 to 115 ng/dL) (reference: 72-153 ng/dL). Patients #6 and #7 normalized TSH. In patient #7, adding iodine allowed PTU dose reduction from 600 to 250 mg/d. At the completion of iodine therapy, FT4 elevation recurred in patients #2 and #3. In patient #5, TSH suppression recurred but the thyroid hormone levels remained normal. Patient #1 remained well-controlled on iodine until undergoing surgery. Patient #6, who continues on iodine monotherapy, has remained well-controlled. No significant difference in FT4 response was observed between SSKI and Lugol’s solution groups (*t* test *P* = 0.961).

Five patients underwent total thyroidectomy (patients #1, #2, #4, #5, #7). Patient #1 sought pregnancy and wished to limit the risk of fetal hypothyroidism; patient #2 experienced iodism and recurrence of thyrotoxicosis; patient #5 was offered surgery due to medication adherence concerns; and patients #4 and #7 pursued surgery to prevent recurrence—for patient #7, surgery was also pursued due to worsening thyromegaly in addition to persistent TSH-suppression on both PTU and iodine add-on. Among the non-surgical patients, patient #3, who was diagnosed with acute myeloid leukemia, discontinued iodine after entering hospice. Patient #6 has remained controlled on iodine monotherapy at least up until the time of article submission.

In total, 71% (5/7) of patients achieved normal thyroid hormone levels and 2/7 (21%) had recurrence of hormone elevations with iodine therapy. Iodine response durations ranged from 20 days to over a year. No adverse events occurred, except for one patient who developed iodism symptoms, including burning sensation in the mouth, pruritis, and nausea (patient #2). These symptoms resolved with a reduction in the iodine dose and supportive care, after which, the patient continued iodine.

## Discussion

In this series, iodine was a feasible treatment option for GD patients who preferred to avoid or could not take thionamides due to contraindications or side effects. Several observational studies from Japan have shown that treating GD with iodine can produce sustained clinical responses, with some experiencing disease remission for over 2 decades after the discontinuation of iodine.^9^, ^10^, ^11^, ^12^, ^13^, ^14^ On average, patients were on iodine for 2 years before they were considered to be in remission.^11^ One propensity score-matched study among treatment-naïve GD patients treated with either MMI or iodine showed that iodine was as effective as MMI at maintaining disease control at 12 months: 85% (17/20) of patients on iodine and 95% (19/20) of patients on MMI achieved normal thyroid function without adverse effects at 1 year.^14^ In the iodine group, 3 patients required add-on thionamide.^14^ In a recent observational study among 288 untreated GD patients followed for 2 years, iodine (starting at 100 mg/d) was effective at controlling disease in >50% of patients, with 43% (123/288) showing sustained response throughout the 2 years.^12^

Treatment response to iodine is typically high (60% to 80%), with a disease remission rate of 40%.^9^ It is unclear which patients are most likely to respond. Age, baseline thyroglobulin and thyroid microsomal antigen autoantibodies, thyroid volume, and free T3, were identified as predictors of good response in one study.^10^ Another study found male sex and higher FT4 levels as predictors of nonresponsiveness.^13^ A review of the Japanese studies found responders were more likely to have milder disease (FT4 <2.76 ng/dL) and smaller goiter, and to be female and elderly.^9^ Interestingly, Thompson et al, in the 1930s had noted that patients with milder GD were more likely to respond than those with more severe disease and larger, firmer, goiters.^2^ Larger studies in the USA and other populations are needed to verify such findings and identify responders versus non-responders. In our series, in terms of final thyroid hormone levels, 2/7 (29%) patients were nonresponders, of which one was acutely ill with leukemia and stopped iodine after transitioning to hospice. Among the 2 nonresponders, one was female, and another was male—both had only a mild thyromegaly. One previous study noted that patients with a small goiter were more sensitive to iodine^10^ while other studies found goiter size predicted neither the response to iodine^9^ nor the recurrence of thyrotoxicosis upon discontinuing iodine.^15^

In patients who do not respond adequately to iodine, several strategies may help improve outcomes.^10^ This may include increasing the dose and/or duration of iodine therapy, adding low-dose thionamide (if not contraindicated), or desensitizing to MMI or PTU.^16^ If these approaches fail, it may be necessary to reconsider surgery or RAI. Preparation for surgery could include a brief course of steroids, beta-blockade, the addition of cholestyramine, and, depending on the degree of thionamide intolerance, a few intermittent doses of thionamide may be tolerated.^17^, ^18^, ^19^ A temporary pause of iodine for at least 2 weeks, and reintroduction approximately 10 days prior to surgery, may restore sensitivity to iodine for preoperative thyroid hormone lowering.

Certain contraindications and safety concerns should be considered when using iodine. We recommend avoiding iodine in patients with prior iodine sensitivity, dermatitis herpetiformis, or hypocomplementemic vasculitis.^20^ Iodism can occur and typically presents with mucocutaneous symptoms such as stomatitis, headache, altered taste, skin lesions, increased salivation and lacrimation, and gastrointestinal discomfort. Long-term hypothyroidism is a possibility, particularly among at-risk patients (those with prior thyroiditis or RAI exposure);^21^^,^^22^ one study noted hypothyroidism development in 5 of 29 (∼17%) of iodine-treated patients.^11^ Furthermore, caution could be exercised when extrapolating findings from iodine-sufficient regions to iodine-insufficient regions. Much of the evidence on the use of iodine for GD comes from Japan, where the daily average intake of iodine is relatively high (1-3 mg/d).^23^ Sudden iodine excess in individuals with long-standing iodine deficiency may increase the risk of thyrotoxicosis.^24^ Lastly, iodine may cause hypothyroidism necessitating thyroid hormone replacement (“block and replace”); however, this effect appears temporary, and replacement is unnecessary for those in remission off iodine therapy.^11^ In one larger study, the incidence of hypothyroidism following iodine ranged from 1% to 11%; the most responsive patients (iodine-sensitive group) had higher occurrences of hypothyroidism.^10^

Several important caveats should be noted in this case series. In patient #3, who was receiving chemotherapy alongside iodine, it is not possible to definitively determine whether the thyrotoxicosis resolved due to iodine or the immunosuppressive effects of chemotherapy. Long-term follow-up of similar patients undergoing both iodine treatment and chemotherapy would be helpful to further explore this. Additionally, in patients who experienced a recurrence of thyroid hormone elevation, we cannot conclusively differentiate whether this was due to thyroiditis or escape from the Wolff-Chaikoff effect. High iodine exposure in these cases precludes RAI uptake scanning; however, monitoring thyroglobulin levels could be informative, especially if there is an abrupt increase in the setting of recurrence, though elevated levels can also be seen in GD. The ratio of FT3 to FT4 may also serve as a useful marker to distinguish between etiologies. For patient #3, who had been on MMI for >18 months prior to iodine treatment, we cannot definitively attribute the initial response to iodine alone, as the residual antithyroid and immunomodulatory effects of long-term thioamide therapy may have contributed. Lastly, we did not systematically monitor iodine adherence and instead relied on patient-reported current use during follow-up visits. Measuring urinary iodine levels could be a more objective method to confirm adherence.

In summary, our experience supports the use of oral iodine (SSKI and Lugol’s) as a treatment option for managing GD. While effective treatments for GD exist, the use of iodine, introduced over 100 years ago, still appears to have merit today, and for some patients it may be the most appropriate choice. Iodine has a low side effect profile and appears to be a viable option for patients with GD who are unable or unwilling to undergo RAI, thyroidectomy, or who cannot take thionamides. Additionally, iodine could serve as a longer-term bridge to surgery. More research is needed to help refine patient selection, doses of iodine needed, and treatment durability, particularly outside of Japan, so that more general recommendations can be made for routine clinical practice.

## Patient Informed Consent

Patients provided informed consent for the publication of this manuscript.

## Disclosure

The authors have no conflicts of interest to disclose.

## References

[1] Starr P., Walcott H.P., Segall H. (1924). The effect of iodin in exophthalmic goiter. JAMA Intern Med.

[2] Thompson W.O., Thompson P.K., Brailey A.G. (1930). Prolonged treatment of exophthalmic goiter by iodine alone. Arch Intern Med.

[3] Jackson A.S. (1925). The effect of the administration of iodine upon exophthalmic goitre: a study of seventy cases thus treated. Ann Surg.

[4] Bloomfield A.L. (1957). The history of the use of iodine in toxic diffuse goiter (Graves' disease). A.M.A.. Arch Intern Med.

[5] Ross D.S., Burch H.B., Cooper D.S. (2016). 2016 American thyroid association guidelines for diagnosis and management of hyperthyroidism and other causes of thyrotoxicosis. Thyroid.

[6] Wolff J., Chaikoff I.L. (1948). Plasma inorganic iodide as a homeostatic regulator of thyroid function. J Biol Chem.

[7] Kopp P.A. (2023). Iodine in the therapy of Graves' disease: a century after Henry S. Plummer. Thyroid.

[8] Wolff J., Chaikoff I.L. (1948). The inhibitory action of iodide upon organic binding of iodine by the normal thyroid gland. J Biol Chem.

[9] Watanabe N. (2025). A narrative review of long-term inorganic iodine monotherapy for Graves' disease with a historical relationship between iodine and thyroid. Endocr J.

[10] Okamura K., Sato K., Fujikawa M. (2022). Iodide-sensitive graves’ hyperthyroidism and the strategy for resistant or escaped patients during potassium iodide treatment. Endocr J.

[11] Okamura K., Sato K., Fujikawa M. (2014). Remission after potassium iodide therapy in patients with Graves’ hyperthyroidism exhibiting thionamide-associated side effects. J Clin Endocrinol Metab.

[12] Fujikawa M., Okamura K. (2024). Graves' hyperthyroidism treated with potassium iodide: early response and after 2 years of follow-up. Eur Thyroid J.

[13] Suzuki N., Yoshimura Noh J., Sugisawa C. (2020). Therapeutic efficacy and limitations of potassium iodide for patients newly diagnosed with Graves’ disease. Endocr J.

[14] Uchida T., Goto H., Kasai T. (2014). Therapeutic effectiveness of potassium iodine in drug-naive patients with Graves’ disease: a single-center experience. Endocrine.

[15] Takata K., Amino N., Kubota S. (2010). Benefit of short-term iodide supplementation to antithyroid drug treatment of thyrotoxicosis due to Graves’ disease. Clin Endocrinol (Oxf).

[16] Mazhari A., Emanuele M.A., Espiritu B. (2021). Desensitization to methimazole. Endocr Pract.

[17] Ozawa Y., Daida H., Shimizu T. (1983). Rapid improvement of thyroid function by using glucocorticoid indicated for the preoperative preparation of subtotal thyroidectomy in Graves’ disease. Endocrinol Jpn.

[18] Baeza A., Aguayo J., Barria M. (1991). Rapid preoperative preparation in hyperthyroidism. Clin Endocrinol (Oxf).

[19] Fischli S., Lucchini B., Muller W. (2016). Rapid preoperative blockage of thyroid hormone production/secretion in patients with Graves’ disease. Swiss Med Wkly.

[20] Sicherer S.H. (2004). Risk of severe allergic reactions from the use of potassium iodide for radiation emergencies. J Allergy Clin Immunol.

[21] Okamura K., Sato K., Fujikawa M. (2023). Painless thyroiditis mimicking relapse of hyperthyroidism during or after potassium iodide or thionamide therapy for Graves’ disease resulting in remission. Endocr J.

[22] Vagenakis A.G., Braverman L.E. (1975). Adverse effects of iodides on thyroid function. Med Clin North Am.

[23] Zava T.T., Zava D.T. (2011). Assessment of Japanese iodine intake based on seaweed consumption in Japan: a literature-based analysis. Thyroid Res.

[24] Sohn S.Y., Inoue K., Rhee C.M. (2024). Risks of iodine excess. Endocr Rev.

